# Accuracy of the digital workflow for guided insertion of orthodontic palatal TADs: a step-by-step 3D analysis

**DOI:** 10.1186/s40510-022-00423-6

**Published:** 2022-08-15

**Authors:** Lucia Pozzan, Marco Migliorati, Luca Dinelli, Riccardo Riatti, Lucio Torelli, Roberto Di Lenarda, Luca Contardo

**Affiliations:** 1 Department of Medical, Surgical and Health Sciences, University of Trieste, Piazza Ospitale 1, 34129 Trieste, Italy; 2 Orthodontic Department, Dental School, University of Genoa, Genoa, Italy

**Keywords:** Miniscrew, Digital workflow, CAD/CAM, Guided surgery, Skeletal anchorage

## Abstract

**Background:**

The introduction in the orthodontic field of the digital workflow for guided insertion of palatal TADs and the development of the 1-visit protocol led to the reduction of chair time and the possibility of complete customization of designs and materials. Conversely, the reduction of operative steps implicates a lower tolerance of deviations between the planned and the actual position of the miniscrews, particularly when the orthodontic device is fixed on 4 palatal TADs or has a rigid structure. This study aims to analyze the influence of each step of the digital workflow on the deviation of the miniscrews’ axis of insertion in a bicortical sample. The null hypothesis is that there are no significant differences in the deviations among the operative steps.

**Methods:**

33 subjects were selected for insertion of bicortical palatal miniscrews with a 1-visit protocol. Digital files were collected at the three stages of the workflow (i.e., digital planning, laboratory prototype, post-insertion impression). A 3D software analysis was performed on a total of 64 miniscrews. After automatic shape recognition of the guiding holes of the digital plan and the scanbodies of the laboratory prototype and post-insertion impression as geometric cylinders, their three-dimensional longitudinal axis was traced and the deviation among them was calculated. Friedman test with Bonferroni correction was performed to assess the significance of the deviations among the three steps, with significance set at *p* < 0.05.

**Results:**

The laboratory step has a significantly lower degree of deviations (2.12° ± 1.62) than both the clinical step (6.23° ± 3.75) and the total deviations (5.70° ± 3.42). No significant differences were found between miniscrews inserted on the left or the right side.

**Conclusions:**

This study suggests that laboratory procedures such as surgical guide production or rapid prototyping don’t play a significant role in the degree of deviations between the planned and the positioned palatal TADs. Conversely, the clinical steps have a bigger influence and need to be carefully evaluated. Despite this difference, there is a cumulative effect of deviations that can lead to the failure of the 1-visit protocol.

## Background

The implementation of new digital technologies in the orthodontic field gave a significant contribution to many aspects of both clinical practice and research, from diagnosis to treatment planning and outcome assessment [1]. One of the main imports of this technological advance was the digitalization of guided procedures for the insertion of palatal TADs. Pre-operative planning and the use of surgical guides allow for precise and controlled placement while minimizing risks associated with this procedure [2, 3].

Many studies support the evidence that pre-operative planning and surgical guides allow for more accurate placement [4–6]. Numerical values on deviations of palatal miniscrews between the planned and the post-operative position vary greatly among studies, and only a few specifically investigate angular deviations between the axis of insertion. Results are generally not directly comparable, because the methods of investigation, the software used, and the reference points analyzed are heterogeneous. What’s more, only a few of these studies are clinical studies, while most of them are cadaveric studies [7] or studies on phantoms [8]. As a general overview, angular deviations in the literature vary from 4.60° ± 2.54° to 3.67° ± 2.25° and 3.60° ± 2.89° [2, 4, 5].

Accuracy between the planned and the actual position of palatal miniscrews has a key role when the 1-visit protocol is applied. The positioning of both the miniscrews and the orthodontic device in the same appointment has many advantages, such as the reduction of chair time and operative steps [9–11]. Many case reports describe the application of this protocol, but studies with larger samples are needed to establish the efficiency and the applicability of these techniques in the daily practice. These studies report some complications ascribable to the misfit between the planned and the positioned miniscrews but fail to quantify them [9–13].

Another advantage of pre-operative planning with a cone-beam computed tomography is the possibility of planning a bicortical position. Bicortical miniscrews show greater stability, better mechanical results, lower stress and strain values, decreased deformation and fracture [14–18]. Despite the number of studies describing the advantage of a bicortical insertion, studies analyzing the deviations between the planned and positioned TADs often fails to indicate whether insertion was monocortical or bicortical. To the best of our knowledge, no clinical investigations specifically focus on deviations between the planned and positioned miniscrews when the lower nasal cortical is engaged.

Considering the heterogeneity of studies on the accuracy of miniscrews placement and how this can influence the 1-visit protocol, there’s the need for a thorough evaluation of accuracy at every step of the digital workflow. To the best of our knowledge, no study analyzes the influence of deviations generated during laboratory processes involved in this specific protocol.

Literature on medical prototyping describes a range of errors varying from 0.13 to 0.57 mm, all considered within clinically acceptable limits [19–22]. Despite the clinical irrelevance of the deviations, investigations highlight how there are potential sources of errors at each stage of the prototyping process[19]. Therefore, even if most studies conclude that inaccuracies in medical rapid prototyping models are unlikely to contribute significantly to errors, they were unable to quantify exactly how much each error source contributes to model accuracy, nor the minimum accuracy required [19–22].

This study aims to test the null hypothesis that there aren’t significant differences in the angular deviations of TADs among each step of the guided digital workflow. The goal is to understand which operative step has the biggest influence on angular deviations between the planned position of palatal miniscrews and the post-insertion position. The experimental approach will be a 3D analysis of digital files corresponding to each of the three stages of the digital workflow (planning, model prototyping, and clinical insertion of the miniscrews). This method has the advantage of allowing a three-dimensional evaluation without the need for adjunctive x-rays exposure. The study will fill in the literature gap by providing an analysis of angular deviations at each stage of the digital workflow and giving novel information on the degree of deviation in a bicortical sample.

## Methods

### Patient selection

Patients in need of orthodontic treatment with a miniscrew-supported palatal appliance were selected from the Section of Orthodontics of the Department of Medicine, Surgery, and Health Sciences of the University of Trieste. The inclusion criteria were the following:

- Indication for a TAD-supported palatal orthodontic device, either with 2, 3, or 4 TADs, including, but not limited to, distalization or mesialization requiring total anchorage, orthopedic palatal expansion in post-pubertal patients, and orthopedic treatment of Class III malocclusions in prepubertal or pubertal patients [23–28].
- Indication for a guided surgical procedure and a digital workflow, including, but not limited to, subjects with anterior crowding, impacted teeth, narrow palate, thick mucosa, and cases where parallelism between the miniscrews is fundamental, such as cases with 4 palatal TADs.

No restrictions were placed regarding the age or gender of the patients considered.

Patients were excluded if they had any systemic disease affecting bone metabolism, syndromes or craniofacial malformations, pathologic processes in the maxilla, use of drugs affecting bone metabolism, compromised immune defense, bleeding disorders, or inadequate oral hygiene [29].

### Digital planning

**Fig. 1:**
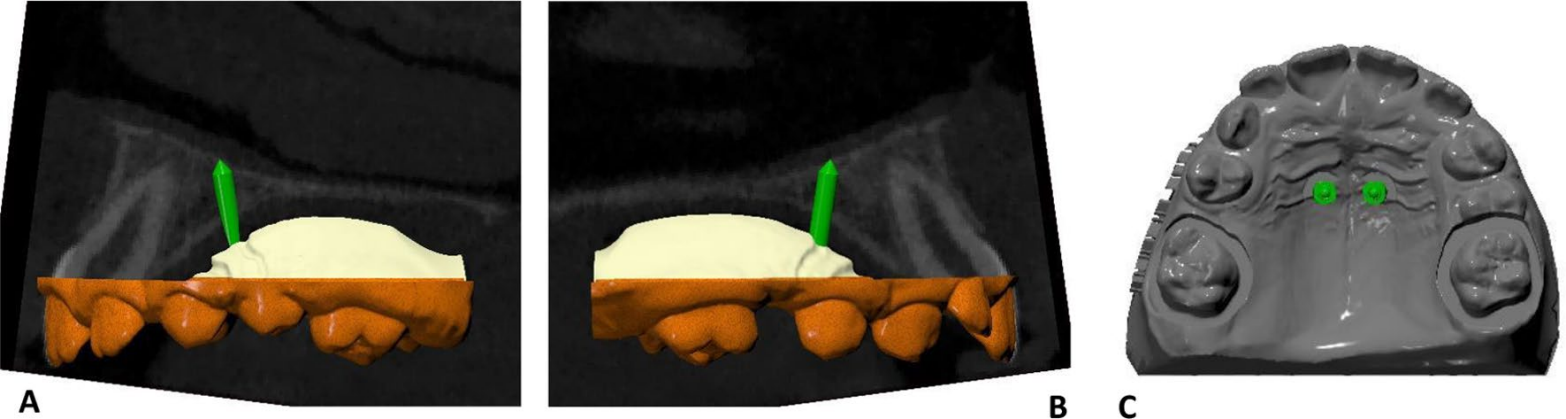
Digital planning of bicortical insertion of two paramedian miniscrews using the REPLICA System®. **A**, **B** virtual miniscrews position on the superimposition of cone-beam computed tomography and digital impression. **C** coronal view of the position of the virtual miniscrews on the digital model

A 1-visit protocol was applied (i.e., insertion of the miniscrews and the orthodontic device in the same appointment), following the REPLICA System®’s planning and insertion protocol (Fig. 1) [6]. In this case, the initial records used are a cone-beam computed tomography (CBCT) (My Ray HyperionX9) and a digital impression of the patient’s upper arch and palate (CS3600, Carestream Dental), that are matched and superimposed with the software Viewbox (dHAL Software, Kifissia, Greece). The same miniscrews (BENEfit®, psm medical solutions) that will be used for the clinical procedure are then selected from a virtual library and positioned based on the bone availability and the future device. Following the guidelines reported in the literature, the correct position of the miniscrews is in the anterior paramedian region, at a 4–5 mm distance from the palatal midline, between the second and third palatal ruga and considering adequate parallelism among the screws and maintaining enough distance from anterior teeth roots. In the posterior palatal region, the premolar and molar areas can be used [7, 8, 30, 31]. The miniscrews are planned in a way to perforate both the palatal and the lower nasal cortical bone (bicortical insertion). Successively, the surgical guide is virtually designed, and guiding pillars and analogs are positioned. The final planning step involves a digital model with holes (named *file 1*) for the actual analogs and the finalization of the surgical guide.

### Laboratory procedure (step 2)

The laboratory step begins with the digital design of the orthodontic device with the software Appliance Designer TM (3Shape A/S, Copenhagen, Denmark). The final step involves the prototyping of the model, the digital impression (CS 3600, Carestream Dental) of the prototype with scan bodies (named file 2), the prototyping of the surgical guide, the sintherization of the orthodontic device, and the fitting of such device on the prototype.

### Surgical procedure (step 3)

After anesthetization with local infiltrative anesthesia, the surgical guide is positioned to check perfect correspondence and stability. The miniscrews used are BENEfit® Orthodontic Screws (PSM Medical Solutions) 2 mm diameter, 9–11 mm length. The procedure was performed with a manually turned unit connected to a contra-angled handpiece (NSK dental). Before that, a pilot hole was performed with a drill equipped with a drill stop calibrated based on the CBCT to perforate only the palatal cortical bone. After positioning the miniscrews, PEEK scan bodies (BENEfit ® system, PSM) are fixed on the screws to acquire a digital impression of their actual position (named *file 3*). The last step involved the fitting of the orthodontic device on the inserted miniscrews.

### Software analysis

**Fig. 2:**
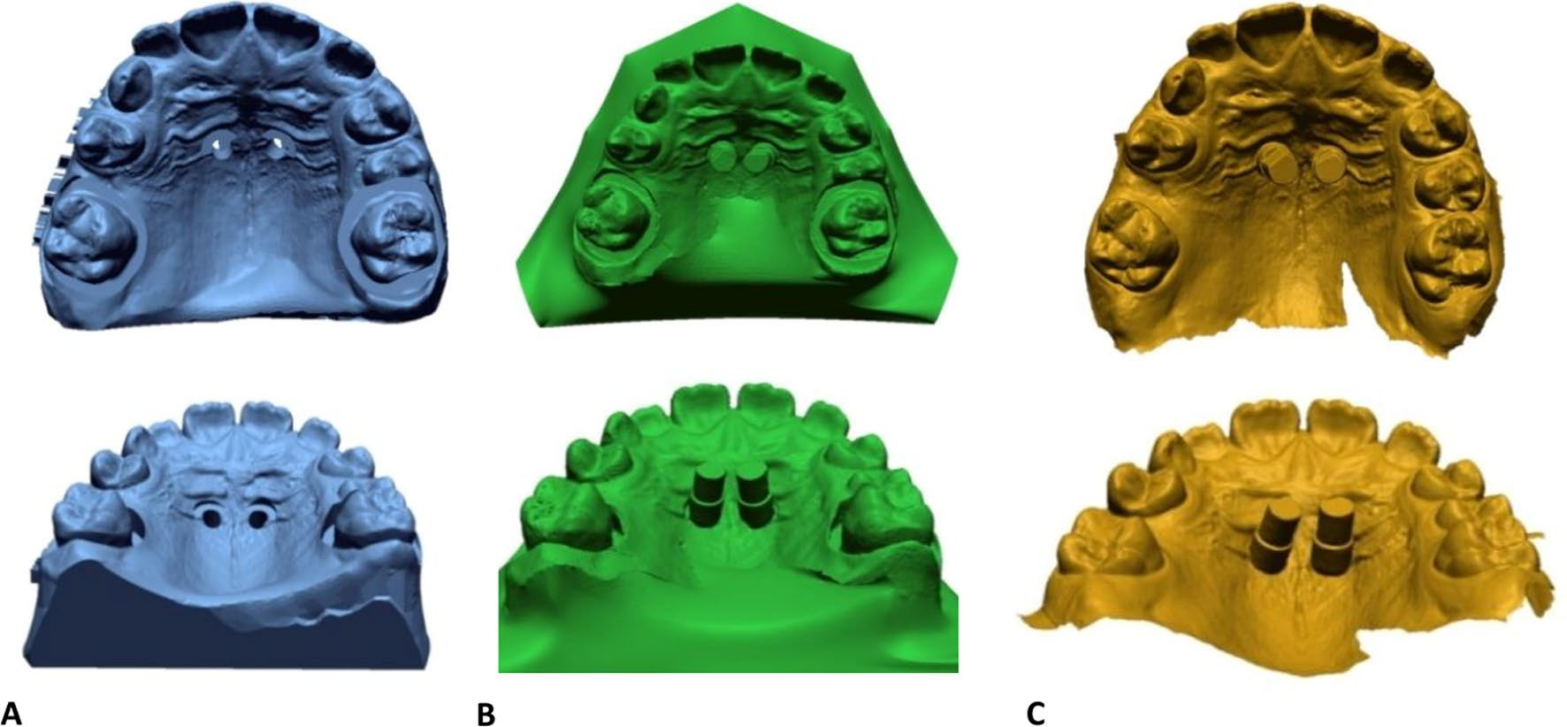
The three files in STL format were analyzed with Geomagic Design X software (Geomagic Design X- version 2019.0.2). **A** digital planning of the miniscrews' position (blue); **B** scanning of the 3D model with scan bodies for the design and fitting of the orthodontic device (green); **C** post-insertion digital impression with scan bodies (yellow)

Software analysis was performed with Geomagic Design X (version 2019.0.2). The three files in STL (Standard Triangle Language) format analyzed for each patient were (Fig. 2):

- *File 1*: the digital plan of the virtual position of the miniscrews (model with holes).
- *File 2*: digital impression with scan bodies of the 3D prototype for fitting of the orthodontic device.
- *File 3*: digital impression with scan bodies of the post-insertion position of the miniscrews after the surgical procedure.

The three files were uploaded and superimposed first roughly with the point-to-point function, choosing the mesiobuccal cusps of the upper first molars and the mesial angle of the incisal edges of the central incisors. A fine superimposition was then performed with an Iterative Closest Point (ICP) algorithm. Once superimposed, an automatic shape recognition function divided each mesh into recognizable and well-defined geometric shapes. At this point, each mesh was visualized singularly, by “switching off” the view of the other two. The following procedure was performed consecutively for the three meshes. The longitudinal axis of the guiding holes (for file 1) and the scan bodies (for files 2 and 3) was drawn with the function “model_add vector_find axis of the cylinder”, selecting the automatically recognized cylinders.

**Fig. 3:**
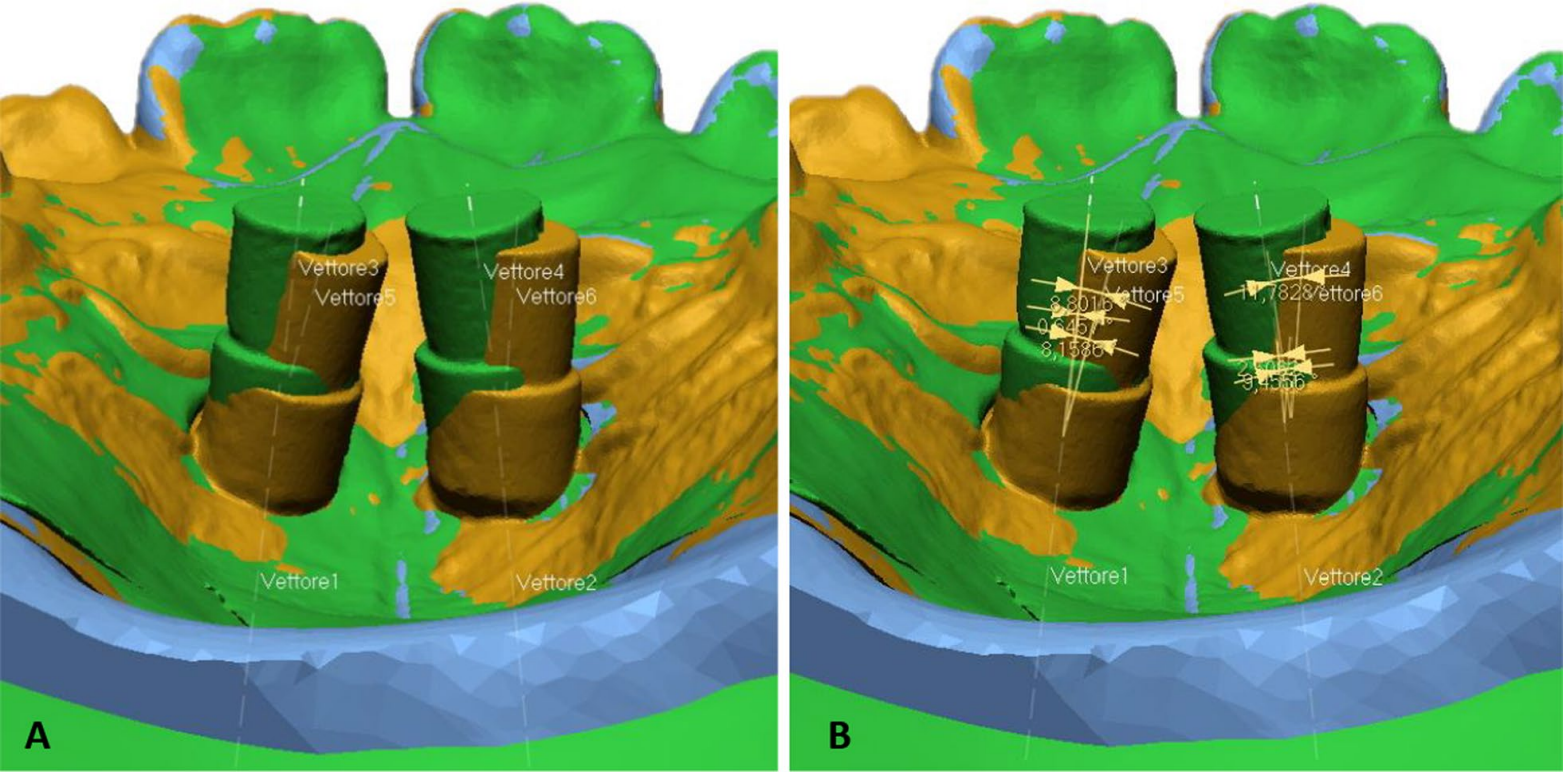
**A** view of all three files is “switched on” once all the axes are identified; **B** angular deviations between vectors are calculated

Once the axes were traced, angular measurements between the digital plan and the laboratory model, the digital plan, and the post-insertion position and between the laboratory model and the post-insertion position were performed with the function “measure angle_ between two vectors” (Fig. 3).

### Error analysis

A subset of 30 randomly chosen measurements was repeated after a 2-week interval by the same investigator. The calibration of the investigator was assessed with the intraclass correlation coefficient (ICC). The ICC for inter-rater reliability was between good and excellent, being 0.93 (0.86–0.97).

### Power analysis

The power analysis found that a sample size of 45 achieve 80% of power to detect a mean of paired differences of 1.5 with a known standard deviation of differences of 3.4 and with a significance level (alpha) of 0.05. Data were acquired from a previous pilot study (unpublished data). A priori sample size required was calculated with G*Power (version 3.1.9.7).

### Statistical analysis

Statistical analysis was performed using the statistical software package SPSS version 26.0 (SPSS Inc., USA). Descriptive statistics were performed and reported as median, IQR range, and range. Mean values ± standard deviations were also reported for uniformity with existing literature. Failure of normality assumption was verified with the Shapiro–Wilk test to yield significant results, thus a non-parametric test for related samples was carried out to test the null hypothesis that there are no significant differences between the deviations among the three operative steps. A Friedman test was performed to compare the deviations among the three operative steps. The significance of the differences in deviations between the left and right sides was tested with the Mann–Whitney U test. Significance was set at *p* < 0.05.

## Results

33 patients were enrolled, 18 females and 15 males. The mean age of the sample was 13.55 years ± 3.46 (14.61 ± 3.5 for females and 12.27 ± 2.99 for males). The total number of miniscrews analyzed was 64, 33 placed on the left side and 31 on the right side. TADs inserted without a thorough following of the protocol (e.g., only partially guided surgical procedures) were not considered, as well as cases with poor quality meshes that couldn’t be univocally analyzed. Table 1 shows the angular deviations between the longitudinal axis of the miniscrew on the digital planning and the laboratory model (named “Laboratory Deviation”), between the laboratory model and the post-insertion position (named “Clinical Deviation”), and between the digital planning and the post-insertion position (named “Total Deviation”) (Table 1).

**Table 1:** Angular deviations (°) of the longitudinal axis

	Lab deviation	Clinical deviation	Total deviation
Mean	2.12	6.23	5.70
SD	1.62	3.75	3.42
Median	1.65	5.30	5.22
IQR	1.76	3.92	3.96
Range	7.69	16.52	16.70

*SD* Standard Deviation; *IQR* interquartile range; *Lab deviation*: the deviation between the digital plan and the laboratory prototype; *Clinical Deviation*: the deviation between the laboratory prototype and the post-insertion position; *Total Deviation*: the deviation between the digital plan and the post-insertion position

The laboratory step, defined as the deviation of the miniscrew’s longitudinal axis between the digital plan and the laboratory prototype, showed a mean deviation of 2.12° ± 1.62; the clinical step, defined as the deviation between the laboratory prototype and the post-insertion position, had a mean deviation of 6.23° ± 3.75°. Finally, the total deviation, defined as the deviation between the digital plan and the intraoral position, was 5.70° ± 3.42.

**Fig.4:**
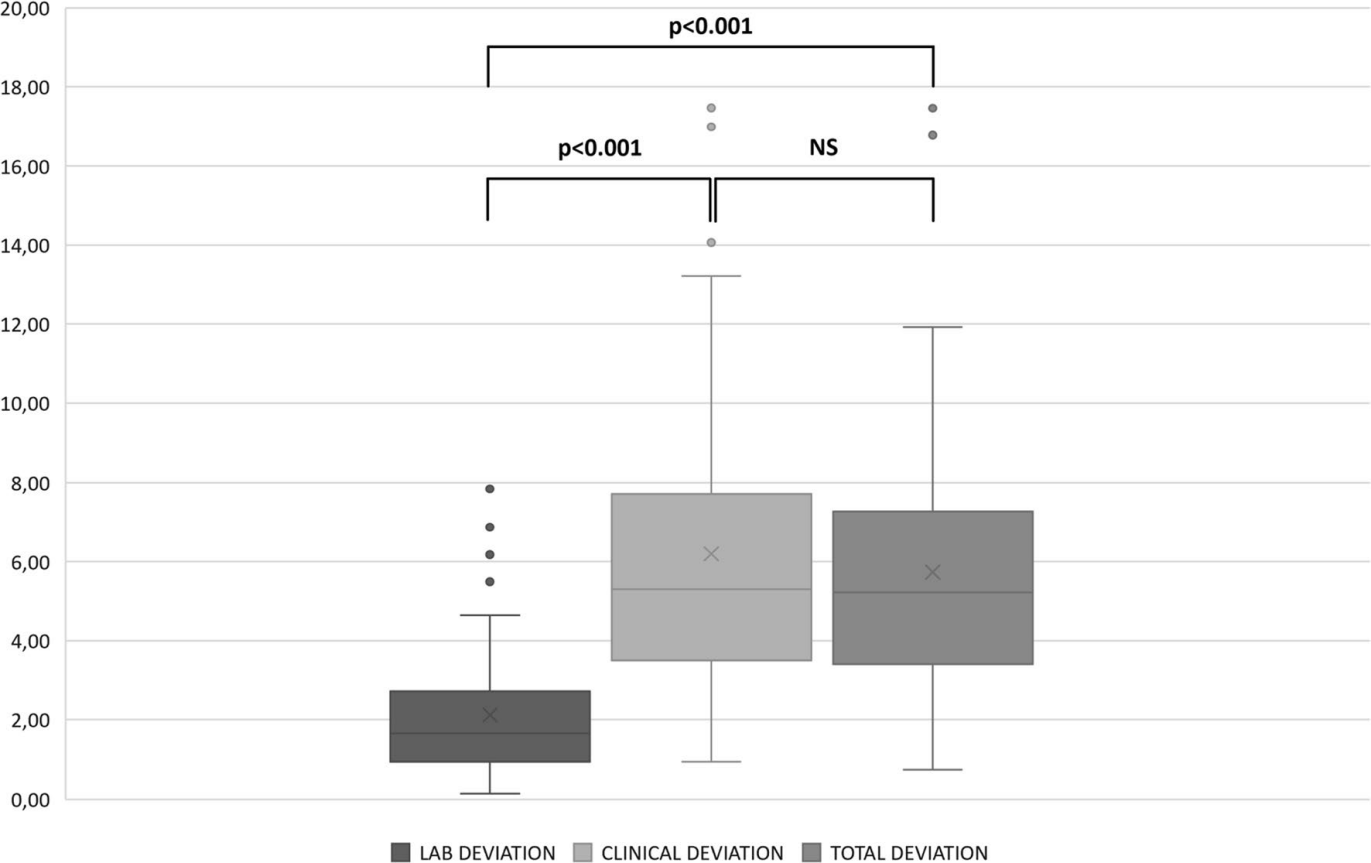
boxplots of the distribution of deviations for the laboratory step, the clinical step, and the total step, and the significance of the differences among the steps (*p* < 0.05)

A significant difference among deviations at each operative step compared to the others was investigated with the Friedman test. There were significant differences between the laboratory deviation and the total deviation (*p* < 0.001), and between the laboratory deviation and the clinical deviation (*p* < 0.001) after Bonferroni adjustments. There were no significant differences between the clinical deviation and the total deviation (*p* = 0.231) (Fig. 4).

No significant differences were found between deviations of the miniscrews inserted on the left and the right side.

## Discussion

This study aimed to investigate the influence of each step of the 1-visit protocol for guided insertion of palatal miniscrews on the accuracy between the planned and the post-insertion position of the miniscrews. We tested the null hypothesis that there are no significant differences regarding the angular deviations of TADs among the three operative steps of the 1-visit protocol.

We found that, in our sample, a certain amount of deviation was inserted at each step of the workflow. In particular, the laboratory step (laboratory deviations: 2.12° ± 1.62) proved to have a significantly smaller influence on the deviation than both the clinical step (clinical deviations: 6.23° ± 3.75) and the total step (5.70° ± 3.42). This result seems to be in line with the literature, showing that the medical prototyping procedure is unlikely to contribute to significant errors [8]. Nevertheless, as small as these laboratory deviations might be, their cumulative effects and their combination with clinical deviations have a strong influence on the clinical practice, particularly when considering a 1-visit protocol. The degree of tolerance for angular deviations decreases as the number of miniscrews, the undercuts, and the rigidity of the orthodontic device increase. If deviations exceed this level of tolerance, the orthodontic device won’t fit properly, and a 2-step protocol must be undertaken [6]. This procedure requires a new digital impression of the actual position of the miniscrews and the alteration of the orthodontic device, and it is not free from inaccuracies and deviations. The use of scan bodies, scanning techniques, and laboratory processes can still introduce some deviations, but the additional impression of the actual position of the miniscrews should guarantee that deviations stay below the tolerance level. That being said, the digital workflow applied to a 1-visit protocol not only has the advantage of reducing the number of appointments but also aims to increase the accuracy by limiting the number of operative steps and the multiple passages between the analogic and the digital workflow [9–12]. Nevertheless, our results show that deviations are far from being clinically irrelevant. Angular values in this study seem to be generally higher than those in the literature, even though results are not directly comparable for the heterogeneity of reference points and measurement methods. No study in the literature clearly describes deviations for a bicortical sample, therefore significant comparisons cannot be made. For the monocortical sample, Möhlhenrich et al. describe deviations up to 6.46° ± 5.5°, a result that is in line with the mean value of our sample [2].

The bigger susceptibility for deviations of the clinical steps may be ascribable to parameters related both to the patient and the clinician [7, 32, 33]. Particularly, characteristics such as bone resistance and density, miniscrews deterioration, and clinical expertise, can have a major influence in bicortical cases, when both the palatal and the lower nasal cortical bone needs to be perforated [34]. A hypothesis that needs to be confirmed with further studies is that contact with the lower nasal cortical bone during bicortical insertion may create an obstacle or a sliding effect that causes the alteration of the insertion path.

Considering the clinical impact of these deviations, it is important to consider that the numerical value of the deviations is not the only parameter that needs consideration. Depending on the number of TADs supporting the palatal device, the direction of the deviations can be in a more favorable configuration, for example when deviations on the paramedian TADs compensate each other, or in a less favorable configuration, e.g., when the direction of deviations is divergent. What’s more, angular deviations can translate into linear deviations happening at the level of the head of the miniscrews, a situation with a great clinical implication for the success of the 1-visit protocol, but also on the tip of the miniscrew and all the intermediate positions between these two.

This study possesses some limitations, thus the clinical theory must be further investigated. Firstly, even though the 3D analysis allows a three-dimensional evaluation of the position of the miniscrews without adjunctive x-ray exposure, it is significantly dependent on the quality of the meshes analyzed. Poor-fitting of the scan bodies or an incorrect scanning technique can directly influence the final analysis. Nevertheless, the literature supports the validity and accuracy of scan bodies to evaluate the position of implants [35]. What’s more, the influence of a bicortical insertion on the degree of deviations must be evaluated through a comparison with a monocortical sample with a comparable workflow.

This study is, to the best of our knowledge, the first one to evaluate the influence of each step of the digital workflow for guided insertion of palatal miniscrews on the accuracy between the planned and final position of the miniscrews. Given the influence of the clinical steps on the angular deviations and the decreasing degree of tolerance of such deviations related to the number of miniscrews and the rigidity of the orthodontic device, the 1-visit protocol is an advisable workflow for cases with 2 TADs, while caution is suggested in cases with 3 or more miniscrews or particularly rigid structure.

## Conclusions

1. All operative steps contribute to a certain degree of deviations that lead to a cumulative effect.
2. The laboratory step has a smaller influence on the angular deviations between planned and inserted miniscrews than the clinical steps.
3. Cases with 2 TADs can be successfully performed with a 1-visit protocol must be approached with caution, given the lower degree of tolerance of the system.

## Data Availability

The dataset used and/or analyzed during the current study are available from the corresponding author on reasonable request.
